# Menteeship as power: Global health must Rethink how it grows its leaders

**DOI:** 10.1371/journal.pgph.0005446

**Published:** 2025-12-30

**Authors:** Ojong Samuel Akombeng, Ngo Bibaa Lundi-Anne Omam, Bate Effim Chloran Louise, Sidney Sangong, Robert Kokou Dowou, Luchuo Engelbert Bain

**Affiliations:** 1 UNICEF Maputo, Maputo, Mozambique; 2 Johns Hopkins Bloomberg School of Public Health, Baltimore, Maryland, United States of America; 3 Reach Out Cameroon, Bakweri Town, Buea, South West Region, Cameroon; 4 Cambridge Public Health Interdisciplinary Research Centre, Department of Psychiatry, University of Cambridge, Cambridge, United Kingdom; 5 Department of Public and Global Health, University of Nairobi, Nairobi, Kenya; 6 Zentrum fur Medizin und Geselschaft, Department of Medical Anthropology, University of Freiburg, Breisgau, Germany; 7 Department of Epidemiology and Biostatistics, FNBSPH, University of Health and Allied Sciences (UHAS), Ho, Volta Region, Ghana; 8 African Population and Health Research Center, Nairobi, Kenya; PLOS: Public Library of Science, UNITED STATES OF AMERICA

## Introduction

Within global and public health practice, emerging professionals navigate cross-cultural, power-laden systems shaped by donor influence, colonial legacies, and entrenched hierarchies that stifle mentorship’s transformative potential [1]. Although often framed as a collaborative alliance for mutual growth [2], mentorship remains largely mentor-centric, concentrating power and success in established figures and fostering dependency on mentor agency, access, and authority. This approach is not tailor-fit for today’s volatile realities. Evidence from low- and middle-income country programs shows that meaningful growth hinges on the mentee’s ability to negotiate these asymmetries [3]. In these settings marked by linguistic diversity, multi-generational teams, and transnational norms, such self-agency is indispensable. We therefore center “menteeship” within the alliance, as a disciplined practice of self-agency, strategic learning, and ethical reflexivity within power-sensitive ecosystems [1]. We also identify common pitfalls, offer a practical toolkit, and outline its operationalization.

## Classic pitfalls and principles of effective menteeship

In global health practice, mentorship often underdelivers because structural and interpersonal pitfalls are amplified by the field’s history and political economy, even as misaligned expectations, vague objectives, and passive menteeship are common [4]. In many LMIC and humanitarian settings, three forces intensify these problems and sustain dependence, weak learning, and inequitable opportunity. First, mentor scarcity and burnout, driven by donor workloads, emergency cycles, and understaffed programs, restrict meaningful access for juniors. Next, entrenched hierarchies between donors and implementers, expatriate and national staff, and Global North and Global South institutions shape who receives guidance and whose contributions are recognized. Furthermore, cultural and identity gradients, including sexism, classism, and ableism, suppress psychological safety and voice, especially for junior women and practitioners from the Global South [5–7].

Evidence underscores that mentorship success depends on the mentee’s proactive agency—clarifying goals, initiating feedback, and sustaining engagement [8,9]. The “25 Principles of Menteeship” codify this shift, promoting mutual respect, empathy, and accountability as tools to counterbalance institutional power [1,5,10]. In practice, global health menteeship requires not only learning from mentors but also co-creating value, challenging extractive dynamics, and shaping equitable exchange aligned with human-centered and decolonial mentorship models [11].

Effective menteeship also requires literacy in organizational culture across governments, UN agencies, NGOs, academic partners, and bilateral programs, where norms and incentives differ sharply. Practitioners must read how hierarchy, funding streams, gender, and identity politics shape guidance, then diversify their mentoring networks to avoid dependence on any single authority. In a plural and power-sensitive ecosystem, such reflexivity is a core professional competency rather than an optional soft skill.

## A competency set for global health mentees

**Fig 1:**
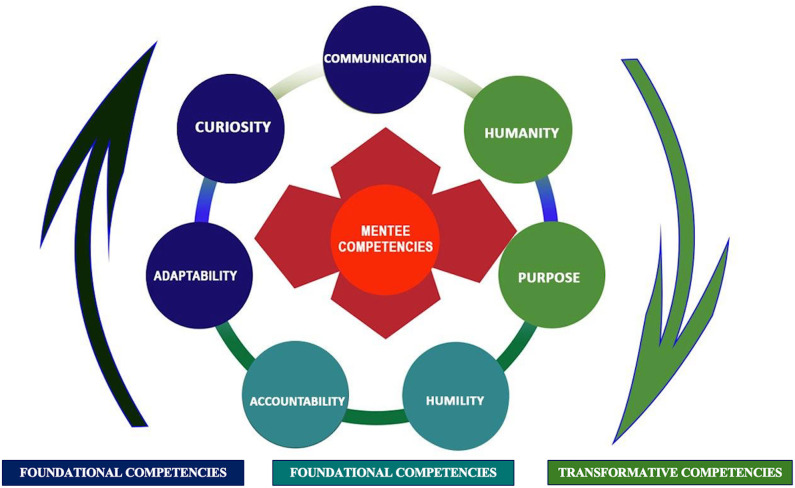
The framework illustrates seven core mentee competencies across three interdependent competency tiers: Foundational (adaptability, curiosity, communication); Relational (accountability, humility); and Transformative (humanity, purpose) required for navigating the unique power structures, cultural complexities, and multi-institutional dynamics of global health ecosystems.

To thrive amid the volatile, uncertain, complex, and ambiguous (VUCA) conditions of global/public health practice, mentees must internalize a cohesive set of interdependent competencies [4]. What distinguishes these competencies within global health from other fields is the need to apply them across transnational contexts, cross-cultural teams, unequal power structures, and fluctuating donor priorities. We therefore propose a three-tier, architecture that centers Foundational, Relational and Transformative competencies (Fig 1).

## Tier 1: Foundational Competencies

These competencies provide the operational literacy required to function across multi-layered global health systems that blend national policy environments, donor requirements, cross-border teams, and culturally diverse communities.

**1. Adaptability** refers to a mentee’s capacity to adjust plans amid uncertainty across emergencies, donor shifts, or political changes, while safeguarding quality [4]. Adaptable mentees pivot to new evidence, resource changes, or emergencies without losing sight of outcomes.
**2. Curiosity** refers to disciplined inquiry as a prerequisite for innovation. It informs the “why/why now,” questions, tests small improvements, and learns iteratively from communities and data [12]. Furthermore, it drives adaptive learning cycles and continuous improvement.
**3. Communication** involves a fine ability to exchange information with clarity and cultural sensitivity [2,10]. In cross-lingual and cross-cultural teams, effective communication also includes navigating translation, power differences, and divergent institutional norms

## Tier 2: Relational Competencies

These competencies build trust and ensure transparency in networks that span governments, donors, academia, and communities, and are critical in a field defined by asymmetry, multiculturalism, and multi-institutional collaboration.

**4. Accountability** is the currency of institutional trust, and addresses a mentee’s commitment to timely delivery, surfaces risks early, and co-defines success metrics with mentors or peers [7]. It includes surfacing risks early, aligning expectations with mentors, and maintaining transparency.
**5. Humility including cultural humility** is especially vital in global health, where practitioners from diverse backgrounds bring different institutional privileges and lived experiences [1]. In global health practice, humility dismantles hierarchical and colonial residues and transforms mentorship into equitable dialogue.

## Tier 3: Transformative Competencies

These competencies anchor practice in meaning, empathy, and justice, and set the tone for ethical practice thus thrusting mentees beyond performance toward shared and transformational leadership.

**6. Humanity** refers to the integrative force that intersects compassion, psychological safety, and the “do-no-harm” ethic into daily work. Furthermore, it ensures that technical excellence remains attached to dignity and care.
**7. Purpose** is the sustained orientation toward people, equity, and systems strengthening rather than personal advancement. It provides resilience during institutional turbulence and aligns individual growth with social impact.

Ultimately, these competencies gain meaning only when embedded within organizational cultures that reinforce them and mentorship structures that model equitable practice.

## A call to action: operationalizing menteeship

The NIH Fogarty International Center embeds structured mentoring across programs such as LAUNCH, Mentoring the Mentors, and Fogarty IeDEA [3,13]. These initiatives show that institutions can formalize mentorship through clear guidance and evaluations. However, in global health where structural inequities and power imbalances persist, operationalizing menteeship requires an expanded vision that clarifies responsibilities for both mentees and mentors.

Mentees should develop structured Individual Development Plans (IDPs) that translate aspirations into SMART skills, milestones, and wellbeing goals [14]. IDPs must reflect local realities including resource constraints to be meaningful [2,5]. In turn, mentors should provide transparent expectations, timely feedback, and equitable access to opportunities. Across diverse institution types, these can be integrated into onboarding routines and HR performance plans and reviewed regularly.

Mentees should engage with a diversified group of mentors who collectively address technical, policy, cultural, and lived-experience needs across global health domains [8,13,15]. Evidence from Fogarty programs in Kenya and Peru shows that group mentoring and peer clusters foster sustainability where senior mentors are scarce [3,15].

All work-based interactions must reflect a power-aware, human-centered ethos [1,11]. Institutions and mentors must center ethics in work credit norms, partnership management, risk flagging and escalation protocols, and the recognition of local expertise. Writing specific ethics clauses into the mentorship compact, addressing scope boundaries, ownership and safe-feedback rules shifts the relationship from extractive to equitable, protecting mentees while reinforcing mentor accountability.

## Conclusion

Reframing menteeship within global health’s historical, political, and structural realities beyond professional development, is a corrective intervention aimed at redistributing power, strengthening equity, and cultivating the next generation of ethical leaders. Because today’s mentee becomes tomorrow’s mentor, institutionalizing menteeship ensures a future in which global health leadership is more just, more accountable, and more respectful of its inherent diversity.
